# Uncovering the phylogeography of *Schinus terebinthifolia* in South Africa to guide biological control

**DOI:** 10.1093/aobpla/plab078

**Published:** 2021-12-23

**Authors:** Kim Canavan, Nwabisa L Magengelele, Iain D Paterson, Dean A Williams, Grant D Martin

**Affiliations:** 1 Centre for Biological Control, Department of Entomology and Zoology, Rhodes University, Makhanda, PO Box 94, 6140, South Africa; 2 Environmental Learning Research Centre, Department of Education, Rhodes University, Makhanda 6140, South Africa; 3 Department of Biology, Texas Christian University, Fort Worth, TX 76129, USA; 4 Afromontane Research Unit and Zoology Department, University of the Free State, Qwaqwa Campus, Phuthaditjhaba 9866, South Africa

**Keywords:** Hybridization, invasive species, microsatellites, phylogeography, *Schinus terebinthifolia*

## Abstract

*Schinus terebinthifolia* is a problematic invasive alien plant (IAP) in South Africa that is a high priority target for biological control. Biological control has been implemented in the states of Florida and Hawaii (USA), where *S. terebinthifolia* is also an IAP. Phylogeographic work determined that there have been multiple introductions of two lineages (haplotype A and B) into the USA. Haplotype A was introduced to western Florida and Hawaii, while haplotype B was introduced to eastern Florida. Haplotypes A and B have subsequently hybridized in Florida, resulting in novel plant genotypes. Biological control agents in the USA are known to vary in efficacies on the two different haplotypes and hybrids. This study used molecular techniques to uncover the source populations of *S. terebinthifolia* in South Africa using chloroplast DNA and microsatellites. Populations from the introduced ranges in Florida (east, west and hybrids) and Hawaii were included (*n* = 95). All South Africa populations (*n* = 51) were found to be haplotype A. Microsatellite analysis determined shared alleles with western Florida and Hawaiian populations. The likely source of South African *S. terebinthifolia* was determined to be western Florida through the horticultural trade. These results will help guide a biological control programme to source agents that perform well on these populations in the USA. Furthermore, the presence of only one haplotype in South Africa highlights the need to ensure no further introductions of other haplotypes of the plant are made, in order to avoid similar hybridization events like those recorded in Florida.

## Teaser

Brazilian peppertree, *Schinus terebinthifolia* is a problematic invasive alien plant in South Africa and is a high priority target for biological control. Previous work in the USA has found that biocontrol agents on *S. terebinthifolia* vary in their efficacy according to plant genotype. This study explored the phylogeography of *S. terebinthifolia* in South Africa and found that only one haplotype is present and it was most likely introduced from western Florida. This work can help guide a biocontrol programme to source agents that perform well on the same haplotype in the USA.

## Introduction

Management of biological invasions can be assisted by knowing the introductory history of a species (Gaskin *et al.* 2011; Hopper *et al.* 2018). Genetic analyses are often the only way to elucidate this phylogeography, as records on the timing and location of introductions are usually lacking or misleading (Estoup and Guillemaud 2010). Phylogeographic studies can also give important insights into the number of independent introductions and their subsequent expansion and patterns of gene flow (Shirk *et al.* 2014). This information can play a crucial role in helping to develop management strategies for invasive alien plants (IAPs), especially for the development of weed biological control (hereafter referred to as biocontrol) programmes (Goolsby *et al.* 2006; Paterson *et al.* 2009; Rollins *et al.* 2009). Such genetic studies have therefore become standard practice in biocontrol programmes (Goolsby *et al.* 2006; Madeira *et al.* 2007; Paterson *et al.* 2009; Paterson and Zachariades 2013; Canavan *et al.* 2017; Kwong *et al.* 2017).

*Schinus terebinthifolia* (Anacardiaceae) (Brazilian peppertree) is a tree native to subtropical South America that has been widely distributed worldwide primarily for its ornamental value (Wheeler *et al.* 2016). The tree has subsequently become invasive in over 20 countries and is considered one of the world’s worst invasive trees (Lowe *et al.* 2000). In the USA, *S. terebinthifolia* has invaded Florida and Hawaii and is one of the most widespread and destructive IAPs in these regions (Schmitz *et al.* 1997; Rodgers *et al.* 2014). A biocontrol programme was initiated in the USA and the pre-introductory evaluations involved genetic work to determine the introductory history of *S. terebinthifolia* (Williams *et al.* 2005). Using nuclear and chloroplast loci it was determined that in Florida there have been multiple introductions of two lineages known as haplotype A and B that originate from different source areas in Brazil (Williams *et al.* 2005, 2007). Haplotype A is predominantly found in western Florida while haplotype B is found in eastern Florida. Intraspecific hybridization between the two haplotypes has subsequently occurred and resulted in novel hybrid genotypes (Williams *et al.* 2005). This has increased genetic variability in the invaded range, and facilitated rapid adaptations to new niches (Williams *et al.* 2007; Geiger *et al.* 2011; Mukherjee *et al.* 2012).

Phylogeographic studies can also assist a biocontrol programme in elucidating how plant genotype may influence biocontrol agent performance. Determination of the evolutionary history of a plant in its invaded range can help lead researchers to the area of origin in the native range that share the same haplotype. Biocontrol agents often have greater performance on target plants that they share an evolutionary history with (Lambert and Casagrande 2007; Bhattarai 2014; Cronin *et al.* 2016). Goolsby *et al.* (2006) demonstrated this sensitivity with populations of an eriophyid mite collected for biocontrol of *Lygodium microphyllum* (Lygodiaceae). Mites collected from plants from the source of the invasive population performed better on the invasive genotype and had reduced fitness and efficacy on plant genotypes from other parts of the native distribution (Goolsby *et al.* 2006). Similarly, the biocontrol programme on *S. terebinthifolia* in the USA has determined that biocontrol agents vary in their efficacies according to plant haplotype (Cuda *et al.* 2012). The three agents (three established in Hawaii and two in mainland USA) were found to have ‘fine tuned’ adaptation to specific populations and genotypes of their hosts plants (Forno 1992; Cuda *et al.* 2012). The occurrence of two different plant genotypes from different source populations and intraspecific hybrids between these genotypes is therefore expected to result in varying levels of control across populations (Manrique *et al.* 2008, 2014; Geiger *et al.* 2011).

*Schinus terebinthifolia* is an IAP in South Africa and is listed as a category 1b species under the National Environmental Management: Biodiversity Act (NEM:BA) (Act 10 of 2004) in the Eastern Cape, Limpopo, Mpumalanga and KwaZulu-Natal provinces and category 3 elsewhere (DEA 2014). In South Africa, *S. terebinthifolia* has invaded across a range of biomes, mostly along the coastal areas of the country and is particularly problematic within the threatened coastal thornveld in the KwaZulu-Natal Province (van der Linden *et al.* 2008; Martin *et al.* 2020).

As biocontrol has already been implemented in the USA, it is considered one of the options for management of *S. terebinthifolia* in South Africa (Byrne *et al.* 2021; Canavan *et al.* 2021). The history and pathways of introduction of S*. terebinthifolia* in South Africa are largely unknown, and since the origin of the invasive populations has proven important for biocontrol in the USA, it is assumed that understanding the origin of the invasive populations in South Africa may be important in selecting effective biocontrol agents. This aim of this study was to determine the introductory history of the invasive populations of *S. terebinthifolia* in South Africa using chloroplast DNA (cpDNA) sequencing and microsatellites.

## Methods

### Study sites

**Figure 1.**
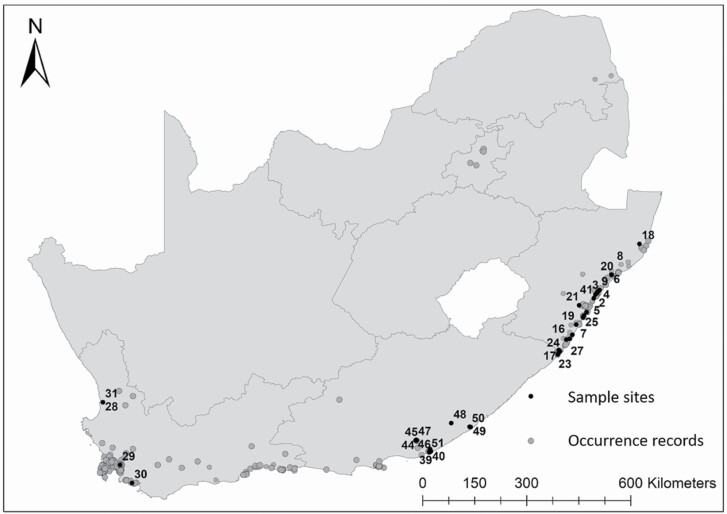
The distribution of sampled populations with corresponding sample numbers **[see****Supporting Information—Table S1****]** and occurrence records showing current distribution of *Schinus terebinthifolia* (records obtained from the Southern African Plant Invaders Atlas (SAPIA, downloaded: 2018) and iNaturalist (downloaded July 2021) (records were filtered to only include Research Grade observations that were indicated to be out of cultivation). WC = Western Cape; EC = Eastern Cape; KZ = KwaZulu-Natal; FS = Free State; MP = Mpumalanga; NC = Northern Cape; GP = Gauteng; NW = North West; LMP = Limpopo Provinces.

Fifty-one trees were sampled from across the plant’s distribution in South Africa (Fig. 1; **see****Supporting Information—Table S1**). A subset (*n* = 95) of the 592 Florida plants analysed in Williams *et al.* (2007) and 45 samples from Hawaii were included **[see****Supporting Information—Table S2****]**. The 95 samples from Florida were chosen to represent the three distinct populations that now occur in that region from the two original introductions of haplotype A into western Florida and haplotype B into eastern Florida (i.e. east, west and hybrid). Samples were selected from STRUCTURE analysis that had the highest shared ancestry with each group (*q*-value) (eastern vs. western *q* > 0.85) as well as hybrids (average *q* in eastern/western introductions = 0.48/0.52) (Williams *et al.* 2007).

### DNA extraction

Fresh leaves were dried in silica gel for all South African samples according to the protocol of Chase and Hills (1991). DNA was extracted using the Qiagen DNeasy® Plant Mini Kit (Valencia, CA, USA) following the manufacturer’s protocol. The protocol was adjusted in that leaf tissue was crushed under liquid nitrogen prior to the extraction. The samples included from Williams *et al.* (2007) were not included in this step as they were received as DNA extracts.

### Sequences

This method was derived from Hamilton (1999) and Williams *et al.* (2005). Chloroplast DNA intraspecific variation was assessed by amplifying the *trnS-trnG* intergenic spacer region **[see****Supporting Information—Table S3****]**. The polymerase chain reaction (PCR) reaction contained 0.4 μL of each of the primers at a concentration of 0.01 μM, 10 μl of Promega Master Mix (Madison, WI, USA), 0.8 μL of Promega MgCl, 3 μL of template DNA per reaction. A further 5.4 μL of Promega nuclease-free water was added to reach a final volume of 20 μL. Amplifications were performed in a T100™ thermal cycler (Bio-Rad, South Africa). The PCR protocol had a 5-min denaturing step at 96 °C followed by 40 cycles of 96 °C for 45 s, annealing temperature of 52 °C for 1 min and 72 °C extension for 30 s. Polymerase chain reaction products were sequenced at Macrogen Corp., Korea using a DNA Analyser 3730x (Applied Biosystems™, Foster City, CA, USA). Samples included from the introduced range (Florida and Hawaii) had already had their haplotypes determined and reported in Williams *et al.* (2005). The cpDNA analysis was therefore only conducted on South African samples.

Chloroplast DNA chromatograms were examined, and contiguous sequences were assembled and manually edited in ChromasLite™ version 2.6.4. An alignment of sequences was done in GeneStudio™ version 2.2.0.0 and included all cpDNA haplotypes for *S. terebinthifolia* that were downloaded from GenBank (accession numbers: AY928398–AY928407 (Williams *et al.* 2005)) with haplotypes confirmed by visual inspection to have all base pairs matched.

### Microsatellites

Microsatellite analysis was performed on the South African samples (*n* = 51) and two samples from Williams *et al.* (2005)**[see****Supporting Information—Table S1****]**. The remaining samples included from Florida and Hawaii were later included with the data set by standardizing the genotypes using the two samples for alignment. Six *S. terebinthifolia* microsatellite loci were grouped into two multiplex reactions according to the protocols of Williams *et al.* (2002, 2005) **[see****Supporting Information—Table S4****]**. Applied Biosystems Inc., UK added fluorescent labels to the primers; stAAG13 with 6-FAM dye, stAAG14 with VIC dye, stGGT39F with NED dye, stCTCCTT01 with PET dye, stAAT1 with 6-FAM dye and stAAT16 with NED dye (DS-33 Matrix Standard (G5 dye set)). The PCR contained ~10 ng of DNA, 12.5 μL of Q5 High-Fidelity 2× Master Mix and 2.5 μL of each primer at concentrations of 10 μM to make a total volume of 25 μL. Reactions were cycled in a Mastercycler® nexus (Eppendorf, Germany), 230 V/50–60 Hz using the simulated tube function. Cycling parameters were one cycle at 98 °C for 30 s, followed by 35 cycles of 10 s at 98 °C, 20 s at 50 °C (multiplex A) or 55 °C (multiplex B), 20 s at 72 °C and a final cycle at 72 °C for 2 min. Capillary electrophoresis was run on an ABI 3500XL (Applied Biosystems™, Foster City, CA, USA) genetic analyser at Inqaba Biotec™.

Chromatogram alignment was first constructed using Geneious version 11.1.5 (Kearse *et al.* 2012). Peak size markers were all aligned to ensure amplified peaks could be aligned by fragment size. The samples from Florida and Hawaii from Williams *et al.* (2007) were then included with the 51 South African samples. The two samples included in the study were used to standardize allelic calls from the results in Williams *et al*. (2005). The data set was entered into a co-dominant matrix and analysed using GenAIEx version 6.5 (Peakall and Smouse 2012).

Observed and expected heterozygosity and *F*_IS_ (inbreeding coefficient) were calculated. We used HP-RARE (Kalinowski 2005) to calculate allelic richness to standardize comparisons across sample sizes. The Hardy–Weinberg equilibrium (HWE) was calculated for each sampling using ‘pegas’ package (version 0.13) in R (Paradis 2010). Genotypic linkage disequilibrium was determined using the genepop package (version 1.1.7) in R (Raymond and Rousset 1995) and the significance threshold was adjusted using Bonferroni correction to account for multiple comparisons (Sokal and Rohlf 1995).

Discriminant analysis of principal components (DAPC) and Bayesian-based STRUCTURE analysis were used to investigate the pattern of population structure. The nuclear ancestry of all 191 samples was tested based on the six microsatellite loci using a Bayesian genetic clustering algorithm implemented in STRUCTURE version 2.3.4. (Pritchard *et al.* 2000). An admixture model was used that assumed correlated allele frequencies, with no location prior with 10 iterations for each run. Each run consisted of 1 000 000 MCMC (Monte Carlo Markov chain) steps and a burn-in period of 50 000. STRUCTURE can give misleading results both for the number of populations and individual ancestry if there is uneven sampling across clusters (*K*; Puechmaille 2016; Wang 2017). We used the recommendations of Wang (2017) and set the prior for admixture to allow *α* to vary between clusters and we decreased the initial *α* from 1.0 to 0.2. We ran 10 independent runs for *K* = 1–5. The most likely *K* was identified using the method of Evanno *et al.* (2005). We then used CLUMPP 1.1.1 (Jakobsson and Rosenberg 2007) to average across the 10 runs for the most likely *K*. Membership assignment of each population was estimated as (*q*), the ancestry coefficient, which varies on a scale from 0 to 1.0, with 1.0 indicating full ancestry with a certain population.

A DAPC analysis was run to complement the STRUCTURE analysis as it is a non-parametric approach and some of the microsatellite loci and populations significantly deviated from the HWE **[see****Supporting Information—Fig. S1****]** (Jombart *et al.* 2010). Discriminant analysis of principal components was used to identify and describe clusters of genetically related individuals, and implemented in the R package adegenet 4.0.2 (Jombart and Ahmed 2011). Discriminant analysis of principal components uses PCA to transform the data to perform a discriminant analysis on the principal components (PCs). This allowed for graphical assessment of the population structure and also calculated the proportion of successful reassignment of individuals to indicate how genetically distinct each population is. A prior step to the DAPC was to determine the optimal number of PCs to retain. We used two methods to calculate this value by predicting the *a-score* and performing a cross-validation method. The *optim.a.score* function (50 replicates *a*-scores were calculated) was used to calculate the maximum *a-score* according to Jombart *et al.* (2010). This measured the discriminating power and stability of DAPC, and was obtained by random permutation of group memberships and comparison of the estimated probabilities of group assignment for the permuted data. The cross-validation approach was performed using the function xvalDapc to fit a DAPC to a subset of the data (a ‘training’ set) and assessing how accurately the model predicts population membership for the samples that were not in the training set. The method that indicated the lowest number of PCs that are sufficient for accurate assignment was used for the subsequent DAPC analysis.

## Results

### Chloroplast DNA

All *S. terebinthifolia* sampled in South Africa (*n* = 51) were determined to be cpDNA haplotype A (GenBank accession number for haplotype A: AY928398). Forty-four samples from Florida were haplotype A and all samples from Hawaii were haplotype A **[see****Supporting Information—Table S2****]**. The remaining samples from Florida were haplotype B, which were mostly found in the east of the State and from hybrid populations. The A and B haplotypes were distinguished by variance at five nucleotide positions out of 716 base pairs (Williams *et al.* 2005).

### Microsatellites

**Figure 2.**
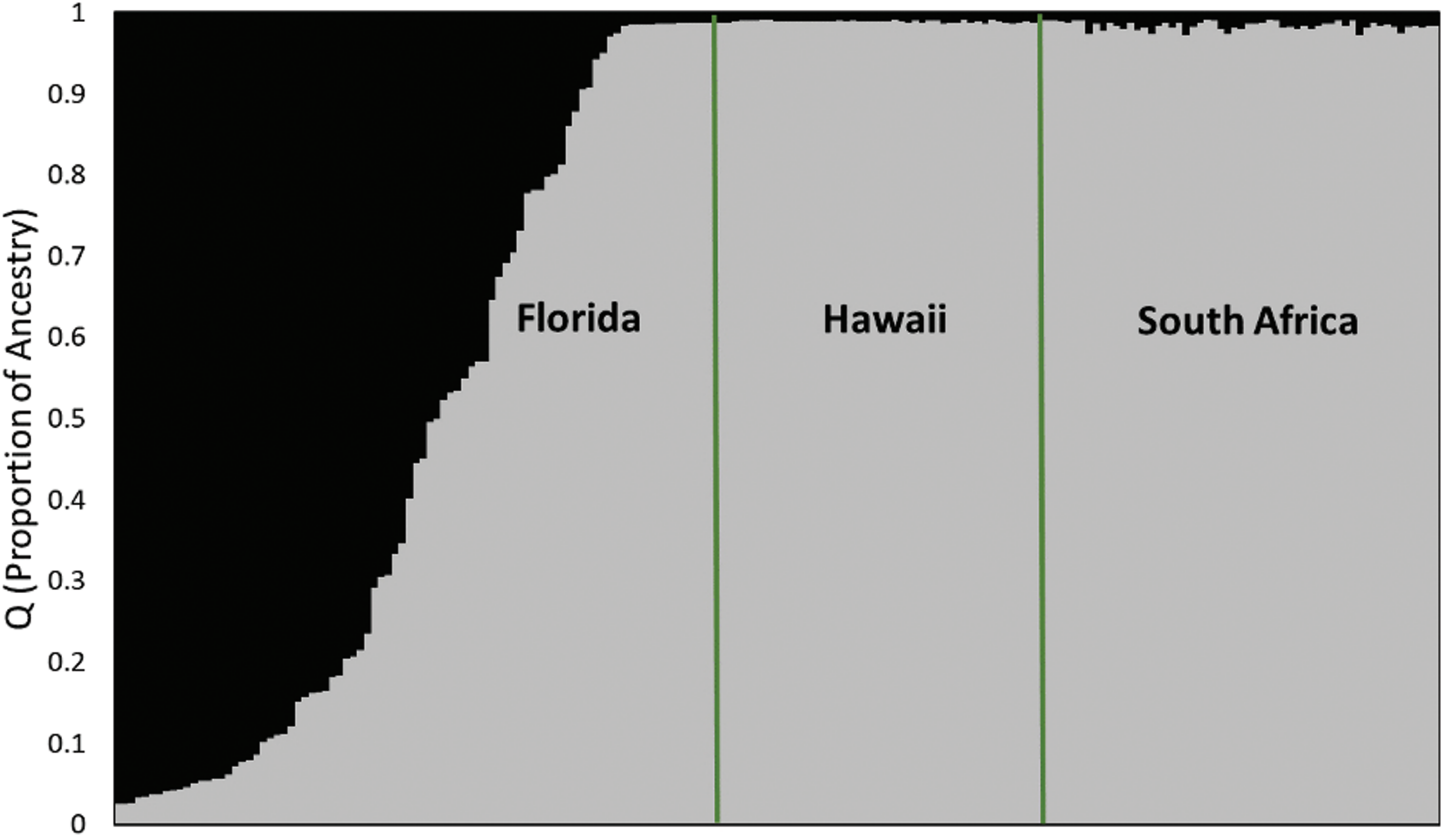
Bayesian clustering of multilocus genotypes from 191 *Schinus terebinthifolia* samples from Florida, Hawaii and South Africa using STRUCTURE (*k* = 2). The proportion of ancestry (*q*) represents the eastern, western and hybrid ancestry of individuals (FL eastern: black; FL western, Hawaii, South Africa: grey).

Based on their microsatellite phenotypes, STRUCTURE HARVESTER analysis determined that all the samples clustered into two populations (*k* = 2) (Fig. 2; **see****Supporting Information—Fig. S2**). Population 1 contained samples from eastern Florida (*n* = 27), and the other cluster was samples from western Florida (*n* = 30), Hawaii (*n* = 45) and South Africa (*n* = 51) (Fig. 2). Hybrid individuals from Florida (*n* = 38) had intermediate values between these two clusters. There was no evidence of hybridization or the presence of hybrids in Hawaii or South Africa.

**Figure 3.**
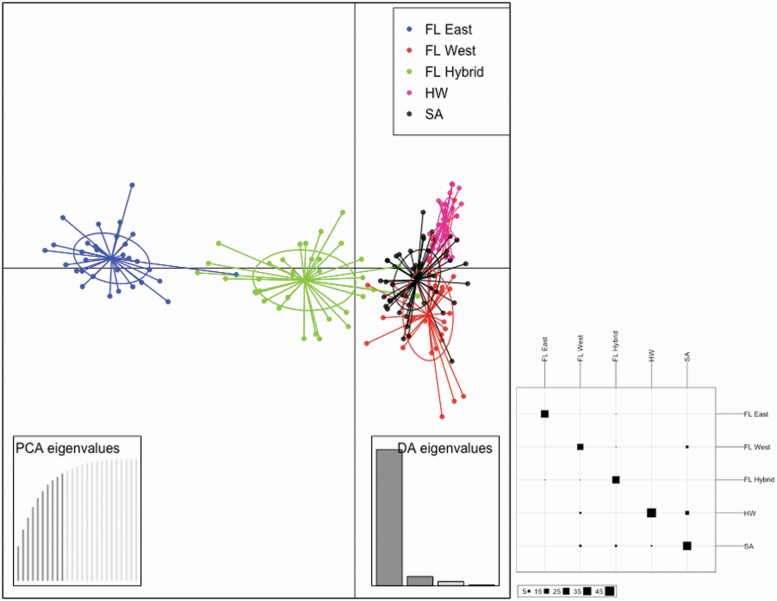
Discriminant analysis of principal components (DAPC) analyses of genotype data for *Schinus terebinthifolia* (*a*-score = 10). Discriminant analysis of principal components was performed using six microsatellite primers (stAAG13, stAAG14, stGGT39F, stCTCCTT01, stAAT1 and stAAT16). Individuals are colour coded based on population (FL East = eastern Florida; FL West = western Florida; FL Hybrid = hybrid populations from east and west Florida; HW = Hawaii; SA = South Africa). A contingency table from the DAPC analysis, utilizing all populations, with columns representing actual clusters of populations and rows representing the inferred clusters based on the predictions of the DAPC analysis (84 % accuracy).

The *a-score* optimization for the DAPC analysis determined the optimum number of PCs was 10 and the cross-validation determined 25 PCs to retain **[see****Supporting Information—Figs S3****and****S4****]**. The *a-score* value indicating the lower number of PCs was used for the DAPC analysis. The DAPC plot outlines the relatedness of the five populations. As with the STRUCTURE analysis the South African, Hawaii and western Florida populations were grouped together (Fig. 3). The South African samples grouped more closely with the western Florida populations compared to the Hawaiian group. The samples from eastern Florida were least genetically similar, grouping away from all other regions. The hybrid populations were found to group between the two main clusters.

South Africa had a total of 15 alleles across all loci and all of these were found in Florida (31 alleles) and in the individuals from western Florida (21 alleles). South African populations were also closely related to the Hawaiian introduction with 11 shared alleles that are also in western Florida.

South Africa had intermediate levels of genetic diversity between Florida and Hawaii (Table 1). The inbreeding coefficient (*F*) was higher in Hawaii compared to Florida and South Africa (Table 1). Observed heterozygosity (*H*_O_) in South African populations averaged 0.46 which was similar to populations from east (*H*_O_ = 0.44) and west ancestry (*H*_O_ = 0.48) in Florida (Table 1). The evaluation of deviation from the HWE determined that three regions had significant deviation, with Hawaiian samples having the most deviation (*P* < 0.05) **[see****Supporting Information—Fig. S1****]**. There was no evidence of significant linkage disequilibrium after Bonferroni correction for multiple comparisons (*P* < 0.003) for any of the locus pairs **[see****Supporting Information—Table S5****]**.

**Table 1.** Mean ± SE genetic diversity measures at six microsatellite loci for *Schinus terebinthifolia* from South Africa, Florida and Hawaii. *N* is the number of individuals in each region; *N*_a_ is the number of alleles; *A*_R_ is allelic richness standardized for 29 individuals; *H*_O_ is observed heterozygosity; *H*_E_ is expected heterozygosity; and *F* is the inbreeding coefficient.

Pop	N	*N*_a_	*A*_R_	*H*_O_	*H*_E_	*F*
South Africa	51	2.50 ± 0.34	2.50 ± 0.34	0.46 ± 0.05	0.48 ± 0.05	0.041
FL East	31	3.83 ± 0.40	3.83 ± 0.40	0.44 ± 0.07	0.52 ± 0.09	0.108
FL West	29	3.50 ± 0.34	3.50 ± 0.34	0.48 ± 0.06	0.54 ± 0.05	0.106
FL Hybrid	35	4.83 ± 0.31	4.71 ± 0.29	0.63 ± 0.04	0.65 ± 0.03	0.017
Hawaii	45	1.83 ± 0.17	1.83 ± 0.17	0.24 ± 0.09	0.31 ± 0.08	0.263

A separate STRUCTURE analysis of only South African samples grouped all the samples into three populations (*k* = 3) **[see****Supporting Information—Figs S5****and****S6****]**. However, the low Delta *K* values (*y*-axis 0–4) indicate that there is no distinct clustering of populations in South Africa. Further, there was no geographic structuring according to allelic phenotypes. Some samples (*n* = 9) shared the same allelic profiles, despite having considerable geographic distances, for example sample 46 (Wilshire, Eastern Cape Province) and 18 (Nyalaza, KwaZulu-Natal Province).

## Discussion

All *S. terebinthifolia* populations surveyed in South Africa were found to be haplotype A and therefore, unlike the invasive populations in Florida, there is no evidence of multiple introductions of genetically distinct lineages. To date, 10 haplotypes of *S. terebinthifolia* have been recorded in the native range (Williams *et al.* 2005). Haplotype A which has been recorded from the native range in Balneário Camboriú, Santa Catarina in southeast Brazil has been determined to be the most common haplotype in the introduced ranges in Hawaii, Florida, Texas, U.S. Virgin Islands (Williams *et al.* 2005) and now South Africa.

Williams *et al.* (2005) found populations of *S. terebinthifolia* in the native range to be more strongly structured according to geographic distance and this was attributed to more limited seed dispersal compared to the exotic range in Florida. Like in Florida, South African populations were not found to be geographically structured and further there was no clear clustering across all samples. In Florida, this has been attributed to two factors: (i) human-driven movement of plants for the ornamental trade and (ii) the long-distance dispersal of seeds by migrating American robins (*Turdus migratorius*) (Williams *et al.* 2005). These same vectors of seed dispersal are likely to have occurred in South Africa. *Schinus terebinthifolia* has also been intentionally planted throughout the country for ornamental, hedging and shade/shelter purposes (Panetta and McKee 1997; Kuruneri-Chitepo and Shackleton 2011). In addition, four common native frugivorous birds were determined to readily consume the fruits of *S. terebinthifolia* which reduces germination time and enhances dispersal of seeds (Dlamini *et al.* 2018). South African populations were also found to have higher genetic diversity compared to Hawaii (higher levels of heterozygosity, HWE) indicating that they are not constrained by a genetic bottleneck which will further enhance their invasive ability.

In South Africa, Hawaii and western Florida, haplotype A is the dominant cpDNA haplotype. Previous studies have determined that populations in Florida have a subset of microsatellite alleles from source regions in South America and Hawaii (Williams *et al.* 2005). However, South Africa, Texas, the U.S. Virgin Islands and other Caribbean islands have a subset of monomorphic alleles from Florida (Williams *et al.* 2005; unpubl.). It therefore seems probable that Florida was the ‘beachhead’ for introductions into South Africa and the other adventive ranges rather than a separate introduction directly from the native range. The USA is the world’s foremost producer of and market for nursery and floriculture plants (Niemiera and Von Holle 2009), particularly in the post-World War II economic boom which increased demand for commercial shipping of ornamental plants (Reichard and White 2001). *Schinus terebinthifolia* was first recorded in 1919 in South Africa (Potts 1919) and this is around the same time of introductions of the plant into Hawaii (de Freitas *et al.* 2020). These introductions to both Hawaii and South Africa would have occurred before plants in Florida became admixed through hybridization of the two haplotypes (Williams *et al.* 2007; Mukherjee *et al.* 2012).

*Schinus terebinthifolia* is also considered invasive in areas that have not been sampled including Spain, Portugal, Australia, New Zealand and some islands in the Pacific and Indian Ocean (CABI 2021). These areas could conceivably also be sources for the South African introduction although it is not clear if these populations existed when *S. terebinthifolia* was introduced into South Africa. Nevertheless, even if one of these unsampled areas was the source for South African *S. terebinthifolia*, this study suggests that they probably would have been genetically very similar to the Florida introduction and so our general conclusions do not change.

The presence of only one source population for *S. terebinthifolia* in South Africa has important implications for management of the plant. Without the presence of additional genotypes in the country there is no potential for admixture or hybridization. Given that hybrids have the potential to have novel phenotypes that can result in higher survival, growth rates and biomass (Geiger *et al.* 2011), it is imperative that legislation is maintained so that no further introductions of *S. terebinthifolia* are allowed into the country that may be the source of new genotypes.

*Schinus terebinthifolia* has not yet had a biocontrol programme initiated in South Africa. A native herbivore, *Megastigmus transvaalensis* (Hymenoptera: Torymidae), uses the plant as a host but has not been found to be suitably damaging to the seeds to impact populations and therefore additional agents are required (Magengelele and Martin 2021). A recent study to determine which IAPs to target for biocontrol in South Africa ranked *S. terebinthifolia* within the top 20 species (out of 299) that are high priority targets (Canavan *et al.* 2021). Brazilian peppertree would represent a transfer project for South Africa given that biocontrol has been implemented elsewhere and agents are available to be directly sourced for host specificity testing (Byrne *et al.* 2021). The outcome of this genetic study will help guide the selection of the most appropriate biocontrol agent.

The absence of any hybridization of haplotypes and comparatively lower genetic diversity than in Florida and the native range enhances the likelihood of achieving successful biocontrol. Plants that have been changed through hybridization and artificial selection are often more difficult to control using biocontrol as they can differ from the plants in the native range where natural enemies have evolved (Paterson *et al.* 2009; Urban *et al.* 2011). This has been the case for biocontrol efforts in Florida whereby the attributes of hybrid populations have resulted in varying levels of control across populations depending on genotype. For example, the introduced seed parasitoid, *M. transvaalensis*, was found to perform poorly on hybrids in Florida (Geiger *et al.* 2011). Furthermore, variation between haplotypes in *S. terebinthifolia* has been found to influence biocontrol agent efficacy. Populations of the recently released, *Pseudophilothrips ichini* (Thysanoptera: Phlaeothripidae) from two geographic locations in Brazil—Ouro Preto, Minas Gerais state (Ouro Preto thrips) and Salvador, Bahia State (Salvador thrips)—differed in their impact on the two haplotypes in Florida (Manrique *et al.* 2014).

## Conclusions

This study determined that populations from western Florida are the likely source of *S. terebinthifolia* in South Africa. Uncovering this pathway of introduction has important implications for management of the plant. Firstly, the presence of only one genotype in the country ensures that hybridization cannot occur and stringent regulation of the plant should continue to ensure new genotypes are not brought in through the horticultural trade. Secondly, the prospects for biocontrol are improved whereby agents can be sourced from populations that have established on genetically similar populations in Florida. The biocontrol agent *P. ichini* has two source populations—‘Ouro Preto’ and ‘Salvador’. The Ouro Preto thrips are likely to be best suited for South Africa as they were sourced off the same haplotype (A) and shown to perform better on *S. terebinthifolia* populations in Florida compared to the Salvador thrip (Manrique *et al.* 2014). In addition to the already established agents in Hawaii and Florida, there are a number of potential biocontrol agents that are likely to be released in the near future (Wheeler *et al.* 2016), including the stem boring weevil, *Apocnemidophorus pipitzi* (Coleoptera: Curculionidae) (Cuda *et al.* 2021), *Calophya lutea* and *C. terebinthifolia* (Hemiptera: Calophyidae) (Prade *et al.* 2021) and *Calophya latiforceps* (Hemiptera: Calophyidae) (Bhattarai *et al.* 2017). These agents should be considered in South Africa and the choice of which agent to use should be guided by climatic matching (Martin *et al.* 2020) and success of establishment on the correct haplotype in Florida.

## Supporting Information

The following additional information is available in the online version of this article—

**Table S1.** Sampling sites for the collection of *Schinus terebinthifolia* genetic material.

**Table S2.** Haplotype information and microsatellite genotype data for all samples including the subset (*n* = 95) of the 592 Florida plants analysed in Williams *et al.* (2007) and 45 samples from Hawaii.

**Table S3.** Primer sequences used to obtain *Schinus terebinthifolia* haplotypes.

**Table S4.** Primer sequences used in this study and their allelic size range for six microsatellite loci of *Schinus terebinthifolia.*

**Table S5.** *P*-values for linkage disequilibrium of each pair of loci across all populations (Fisher’s method). Using the Bonferroni correction (*P <* 0.003) there were no significant deviations.

**Figure S1.** Heat map showing *P*-values of deviations from the Hardy–Weinberg equilibrium (HWE) across populations and marker (*P* <= 0.05). FL East = East Florida, FL West = West Florida, FL Admixed = hybrid populations in Florida, HW = Hawaii, SA = South Africa.

**Figure S2.** Delta *K* values showing the ideal number of populations as *k* = 2 based on 191 samples of *Schinus terebinthifolia* from South Africa, Hawaii and Florida using six microsatellite primer pairs and the Evanno method implemented in STRUCTURE HARVESTER program according to Earl and von Holdt (2012).

**Figure S3.** The *a-score* optimization showing the optimum number of retained principal components (PCs) is 10.

**Figure S4.** The discriminant analysis of principal components (DAPC) cross-validation showing the optimum number of retained principal components (PCs) is 25.

**Figure S5.** Genetic population structure of 51 individuals of *Schinus terebinthifolia* from populations in South Africa, based on Bayesian clustering analysis of microsatellite loci with STRUCTURE (Pritchard *et al.* 2000). According to the Evanno method, three populations were inferred **[see****Supporting Information—Fig. S6****]**. Sample ordered according to *Q*-value. The red cluster corresponds to population 1, the green cluster corresponds to population 2 and blue corresponds to population 3. The values in ordinate the shared ancestry according to percentage membership into each population.

**Figure S6.** Delta *K* values showing the ideal number of populations as *k* = 3 based on 51 samples of *Schinus terebinthifolia* from South Africa using six microsatellite primer pairs and the Evanno method implemented in STRUCTURE HARVESTER program according to Earl and von Holdt (2012).

plab078_suppl_Supplementary_Material

plab078_suppl_Supplementary_Table_S2

## Data Availability

All cpDNA haplotype sequences of *Schinus terebinthifolia* used in this study are available from NCBI (https://www.ncbi.nlm.nih.gov/). The GenBank accession numbers are AY928398 (https://www.ncbi.nlm.nih.gov/nuccore/AY928398.1/) and AY928399.1 (https://www.ncbi.nlm.nih.gov/nuccore/61723680). Microsatellite genotype data are available as **Supporting Information—Table S2**.

## Notes

**Evolution & Diversity. Chief Editor**: Jeremy Beaulieu

## References

[CIT0001] Bhattarai GP . 2014. Biogeographical approaches for studying species invasion. PhD Thesis, Louisiana State University, Baton Rouge, LA.

[CIT0002] Bhattarai GP , Diaz R, Manrique V, Turechek WW, Buss L, Stange B, Overholt WA. 2017. Diversity and impact of herbivorous insects on Brazilian peppertree in Florida prior to release of exotic biological control agents. Biocontrol Science and Technology 27:703–722.

[CIT0003] Byrne MJ , Mayonde S, Venter N, Chidawanyika F, Zachariades C, Martin G. 2021. Three new biological control programmes for South Africa: Brazilian pepper, *Tamarix* and *Tradescantia*. African Entomology 29:965–982.

[CIT0004] CABI . 2021. *Schinus terebinthifolius (Brazilian pepper tree).* Centre for Agriculture and Bioscience International. https://www.cabi.org/isc/datasheet/49031 (11 August 2021).

[CIT0005] Canavan K , Paterson ID, Hill MP. 2017. Exploring the origin and genetic diversity of the giant reed, *Arundo donax* in South Africa. Invasive Plant Science and Management 10:53–60.

[CIT0006] Canavan K , Paterson ID, Ivey P, Sutton GF, Hill MP. 2021. Prioritisation of targets for weed biological control III: a tool to identify the next targets for biological control in South Africa and set priorities for resource allocation. Biocontrol Science and Technology 31:584–601.

[CIT0007] Chase MW , Hills HH. 1991. Silica gel, an ideal material for field preservation of leaf samples for DNA studies. Taxon 40:215–220.

[CIT0008] Cronin JT , Kiviat E, Meyerson LA, Bhattarai GP, Allen WJ. 2016. Biological control of invasive *Phragmites australis* will be detrimental to native *P. australis*. Biological Invasions 18:2749–2752.

[CIT0009] Cuda JP , Christ L, Manrique V, Overholt W, Wheeler G, Williams D. 2012. Role of molecular genetics in identifying ‘fine tuned’ natural enemies of the invasive Brazilian peppertree, *Schinus terebinthifolius*: a review. BioControl 57:227–233.

[CIT0011] Cuda JP , Gillmore JL, Garcete-Barrett BR, Benda N, Sharma S. 2021. Is the stem boring weevil *Apocnemidophorus pipitzi* (Coleoptera: Curculionididae) host specific to *Schinus terebinthifolia* (Sapindales: Anacardiaceae)? Biocontrol Science and Technology. doi:10.1080/09583157.2021.1929071.

[CIT0012] de Freitas TC , Guarino ED, Gomes GC, Molina AR, da Luz Real IM, Beltrame R. 2020. The effect of seed ingestion by a native, generalist bird on the germination of worldwide potentially invasive trees species *Pittosporum undulatum* and *Schinus terebinthifolia.* Acta Oecologica 108:103639.

[CIT0013] Department of Environmental Affairs (DEA). 2014. National Environmental Management, Biodiversity Act (NEMBA), 2004 (Act No. 10 of 2004) Alien and Invasive Species Lists, 2014. Government Gazette No. 37886.

[CIT0014] Dlamini P , Zachariades C, Downs CT. 2018. The effect of frugivorous birds on seed dispersal and germination of the invasive Brazilian pepper tree (*Schinus terebinthifolia*) and Indian laurel (*Litsea glutinosa*). South African Journal of Botany 114:61–68.

[CIT0015] Earl DA , von Holdt BM. 2012. STRUCTURE HARVESTER: a website and program for visualizing STRUCTURE output and implementing the Evanno method. Conservation Genetics Resources 4:359–361.

[CIT0016] Estoup A , Guillemaud T. 2010. Reconstructing routes of invasion using genetic data: why, how and so what? Molecular Ecology 19:4113–4130. 20723048 10.1111/j.1365-294X.2010.04773.x

[CIT0017] Evanno G , Regnaut S, Goudet J. 2005. Detecting the number of clusters of individuals using the software STRUCTURE: a simulation study. Molecular Ecology 14:2611–2620. 15969739 10.1111/j.1365-294X.2005.02553.x

[CIT0062] Forno IW, Kassulke RC, Harley KL. 1992. Host specificity and aspects of the biology of *Calligrapha pantherina* (Col.: Chrysomelidae), a biological control agent of Sida acuta [Malvaceae] and *S. rhombifolia* in Australia. Entomophaga 37:409–17.

[CIT0018] Gaskin JF , Bon MC, Cock MJ, Cristofaro M, De Biase A, De Clerck-Floate R, Sforza R. 2011. Applying molecular-based approaches to classical biological control of weeds. Biological Control 58:1–21.

[CIT0019] Geiger JH , Pratt PD, Wheeler GS, Williams DA. 2011. Hybrid vigor for the invasive exotic Brazilian peppertree (*Schinus terebinthifolia* Raddi., Anacardiaceae) in Florida. International Journal of Plant Sciences 172:655–663.

[CIT0020] Goolsby JA , DE Barro PJ, Makinson JR, Pemberton RW, Hartley DM, Frohlich DR. 2006. Matching the origin of an invasive weed for selection of a herbivore haplotype for a biological control programme. Molecular Ecology 15:287–297. 16367847 10.1111/j.1365-294X.2005.02788.x

[CIT0021] Hamilton MB . 1999. Four primer pairs for the amplification of chloroplast intergenic regions with intraspecific variation. Molecular Ecology 8:521–523. 10199016

[CIT0022] Hopper JV , McCue KF, Pratt PD, Duchesne P, Grosholz ED, Hufbauer RA. 2018. Into the weeds, matching importation history to genetic consequences and pathways in two widely used biological control agents. Evolutionary Applications. doi:10.1111/eva.12755. PMC6439500 30976309

[CIT0063] Jakobsson M, Rosenberg NA. 2007. CLUMPP: a cluster matching and permutation program for dealing with label switching and multimodality in analysis of population structure. Bioinformatics 23:1801–6. 10.1093/bioinformatics/btm233 17485429

[CIT0023] Jombart T , Ahmed I. 2011. adegenet 1.3-1: new tools for the analysis of genome-wide SNP data. Bioinformatics 27:3070–3071. 21926124 10.1093/bioinformatics/btr521 PMC3198581

[CIT0024] Jombart T , Devillard S, Balloux F. 2010. Discriminant analysis of principal components: a new method for the analysis of genetically structured populations. BMC Genetics 11:94. 20950446 10.1186/1471-2156-11-94 PMC2973851

[CIT0064] Kalinowski ST. 2005. HP-RARE 1.0: a computer program for performing rarefaction on measures of allelic richness. Molecular Ecology Notes 5:187–9.

[CIT0025] Kearse M , Moir R, Wilson A, Stones-Havas S, Cheung M, Sturrock S, Buxton S, Cooper A, Markowitz S, Duran C, Thierer T, Ashton B, Meintjes P, Drummond A. 2012. Geneious Basic: an integrated and extendable desktop software platform for the organization and analysis of sequence data. Bioinformatics 28:1647–1649. 22543367 10.1093/bioinformatics/bts199 PMC3371832

[CIT0026] Kuruneri-Chitepo C , Shackleton CM. 2011. The distribution, abundance and composition of street trees in selected towns of the Eastern Cape, South Africa. Urban Forestry and Urban Greening 10:247–254.

[CIT0027] Kwong RM , Sagliocco JL, Harms NE, Butler K, Green PT, Martin GD. 2017. Biogeographical comparison of the emergent macrophyte, *Sagittaria platyphylla* in its native and introduced ranges. Aquatic Botany 141:1–9.

[CIT0028] Lambert AM , Casagrande RA. 2007. Susceptibility of native and non-native common reed to the non-native mealy plum aphid (Homoptera: Aphididae) in North America. Environmental Entomology 36:451–457. 17445381 10.1603/0046-225x(2007)36[451:sonanc]2.0.co;2

[CIT0029] Lowe S , Browne M, Boudjelas S, De Poorter M. 2000. 100 of the world’s worst invasive alien species: a selection from the global invasive species database (vol. 12). Auckland, New Zealand: Invasive Species Specialist Group.

[CIT0030] Madeira PT , Coetzee JA, Center TD, White EE, Tipping PW. 2007. The origin of *Hydrilla verticillata* recently discovered at a South African dam. Aquatic Botany 87:176180.

[CIT0031] Magengelele NL , Grant M. 2021. Distribution and impact of the native South African wasp, *Megastigmus transvaalensis* Hussey (Hymenoptera: Torymidae) on the invasive S*chinus terebinthifolia* in South Africa. African Entomology, in press.

[CIT0032] Manrique V , Cuda JP, Overholt WA, Williams DA, Wheeler GS. 2008. Effect of host-plant genotypes on the performance of three candidate biological control agents of *Schinus terebinthifolia* in Florida. Biological Control 47:167–171.

[CIT0033] Manrique V , Diaz R, Erazo L, Reddi N, Wheeler GS, Williams D, Overholt WA. 2014. Comparison of two populations of *Pseudophilothrips ichini* (Thysanoptera: Phlaeothripidae) as candidates for biological control of the invasive weed *Schinus terebinthifolia* (Sapindales: Anacardiaceae). Biocontrol Science and Technology 24:518–535.

[CIT0034] Martin GD , Magengelele NL, Paterson ID, Sutton GF. 2020. Climate modelling suggests a review of the legal status of Brazilian pepper *Schinus terebinthifolia* in South Africa is required. South African Journal of Botany 132:95–102.

[CIT0035] Mukherjee A , Williams DA, Wheeler GS, Cuda JP, Pal S, Overholt WA. 2012. Brazilian peppertree (*Schinus terebinthifolia*) in Florida and South America: evidence of a possible niche shift driven by hybridization. Biological Invasions 14:1415–1430.

[CIT0036] Niemiera AX , Von Holle B. 2009. Invasive plant species and the ornamental horticulture industry. In: Inderjit A, ed. Management of invasive weeds. Dordrecht, The Netherlands: Springer, 167–187.

[CIT0037] Panetta FD , McKee J. 1997. Recruitment of the invasive ornamental, *Schinus terebinthifolia* is dependent upon frugivores. Australian Journal of Ecology 22:432–438.

[CIT0038] Paradis E . 2010. pegas: an R package for population genetics with an integrated-modular approach. Bioinformatics 26:419–420. 20080509 10.1093/bioinformatics/btp696

[CIT0039] Paterson ID , Downie DA, Hill MP. 2009. Using molecular methods to determine the origin of weed populations of *Pereskia aculeata* in South Africa and its relevance to biological control. Biological Control 48:84–91.

[CIT0040] Paterson ID , Zachariades C. 2013. ISSRs indicate that *Chromolaena odorata* invading southern Africa originates in Jamaica or Cuba. Biological Control 66:132–139.

[CIT0041] Peakall R , Smouse PE. 2012. GenAlEx 6.5: genetic analysis in Excel. Population genetic software for teaching and research–an update. Bioinformatics 28:2537–2539. 22820204 10.1093/bioinformatics/bts460 PMC3463245

[CIT0042] Potts G . 1919. The pepper tree (*Schinus molle*) in its relationship to epidemic hayfever: interim report. South African Journal of Science 15:525–530.

[CIT0043] Prade P , Minteer CR, Gezan SA, Arguijo VC, Bowers K, Cuda JP, Overholt WA. 2021. Host specificity and non-target longevity of *Calophya lutea* and *Calophya terebinthifolii*, two potential biological control agents of Brazilian peppertree in Florida, USA. BioControl 66:281–294.

[CIT0044] Pritchard JK , Stephens M, Donnelly P. 2000. Inference of population structure using multilocus genotype data. Genetics 155:945–959. 10835412 10.1093/genetics/155.2.945 PMC1461096

[CIT0045] Puechmaille SJ . 2016. The program structure does not reliably recover the correct population structure when sampling is uneven: subsampling and new estimators alleviate the problem. Molecular Ecology Resources 16:608–627. 26856252 10.1111/1755-0998.12512

[CIT0046] Raymond M , Rousset F. 1995. Genepop (version 1.2) population genetics software for exact tests and ecumenicism. Journal of Heredity 86:248–249.

[CIT0047] Reichard SH , White P. 2001. Horticulture as a pathway of invasive plant introductions in the United States: most invasive plants have been introduced for horticultural use by nurseries, botanical gardens, and individuals. BioScience 51:103–113.

[CIT0049] Rodgers L , Pernas T, Hill SD. 2014. Mapping invasive plant distributions in the Florida Everglades using the digital aerial sketch mapping technique. Invasive Plant Science and Management 7:360–374.

[CIT0050] Rollins LA , Woolnough AP, Wilton AN, Sinclair R, Sherwin WB. 2009. Invasive species can’t cover their tracks: using microsatellites to assist management of starling (*Sturnus vulgaris*) populations in Western Australia. Molecular Ecology 18:1560–1573. 19317845 10.1111/j.1365-294X.2009.04132.x

[CIT0051] Schmitz DC , Simberloff D, Hofstetter RH, Haller W, Sutton D. 1997. The ecological impact of nonindigenous plants. In: Simberloff D, Schmitz DC, Brown TC, eds. Strangers in paradise. Washington, DC: Island Press, 39–61.

[CIT0052] Shirk RY , Hamrick JL, Zhang C, Qiang S. 2014. Patterns of genetic diversity reveal multiple introductions and recurrent founder effects during range expansion in invasive populations of *Geranium carolinianum* (Geraniaceae). Heredity 112:497–507. 24346497 10.1038/hdy.2013.132 PMC3998781

[CIT0053] Sokal RR , Rohlf FJ. 1995. Biometry. New York: Freeman WH.

[CIT0055] Urban AJ , Simelane DO, Retief E, Heystek F, Williams HE, Madire LG. 2011. The invasive ‘*Lantana camara* L.’ hybrid complex (Verbenaceae), a review of research into its identify and biological control in South Africa. African Entomology 19:315–348.

[CIT0056] van der Linden JP , Siebert F, Siebert SJ, Ferreirai DP, Bredenkamp GJ. 2008. Characterisation of the woody assemblages of Zululand coastal thornveld along the Nseleni river. Koedoe 50:113–125.

[CIT0057] Wang J . 2017. The computer program structure for assigning individuals to populations: easy to use but easier to misuse. Molecular Ecology Resources 17:981–990. 28028941 10.1111/1755-0998.12650

[CIT0058] Wheeler GS , Kay FM, Vitorino MD, Manrique V, Diaz R, Overholt WA. 2016. Biological control of the invasive weed *Schinus terebinthifolia* (Brazilian Peppertree), a review of the project with an update on the proposed agents. South Eastern Naturalist 15:15–34.

[CIT0059] Williams DA , Muchugu E, Overholt WA, Cuda JP. 2007. Colonization patterns of the invasive Brazilian peppertree, *Schinus terebinthifolius*, in Florida. Heredity 98:284–293. 17245420 10.1038/sj.hdy.6800936

[CIT0060] Williams DA , Overholt WA, Cuda JP, Hughes CR. 2005. Chloroplast and microsatellite DNA diversities reveal the introduction history of Brazilian peppertree (*Schinus terebinthifolius*) in Florida. Molecular Ecology 14:3643–3656. 16202086 10.1111/j.1365-294X.2005.02666.x

[CIT0061] Williams DA , Sternberg LdaSL, Hughes CR. 2002. Characterization of polymorphic microsatellite loci in the invasive Brazilian peppertree, *Schinus terebinthifolia*. Molecular Ecology Notes 2:231–232.

