# Supplementary material for: Uncovering the phylogeography of Schinus terebinthifolia in South Africa to guide biological control
Source: AoB Plants. 2021 Dec 23;14(1):plab078. doi: 10.1093/aobpla/plab078 (PMC8783615; doi:10.1093/aobpla/plab078)
Supplement: plab078_suppl_Supplementary_Material [file plab078_suppl_supplementary_material.pdf]

**Supporting Information**

**Table S1.** Sampling sites for the collection of *Schinus terebinthifolia* genetic material.

| Sample number | Site location | Province | GPS coordinates (latitude; longitude) |
|---|---|---|---|
| 1 | Shakaskraal | KwaZulu-Natal | -29,4443; 31,23997 |
| 2 | Shakaskraal | KwaZulu-Natal | -29,4524; 31,21672 |
| 3 | R102 | KwaZulu-Natal | -29,5048; 31,18503 |
| 4 | Ballito | KwaZulu-Natal | -29,5041; 31,1944 |
| 5 | Umzinto | KwaZulu-Natal | -30,1261; 30,84441 |
| 6 | Eshowe | KwaZulu-Natal | -29,0121; 31, 57952 |
| 7 | Hibberdene | KwaZulu-Natal | -30,5704; 30,56809 |
| 8 | Eshowe | KwaZulu-Natal | -29,0121; 31,57952 |
| 9 | Dube village | KwaZulu-Natal | -29,412; 31,27277 |
| 10 | Shibumi | KwaZulu-Natal | -30,6956; 30,41586 |
| 11 | Amazimtoti | KwaZulu-Natal | -29,9855; 30,93678 |
| 12 | Port Edward | KwaZulu-Natal | -30,9659; 30, 21387 |
| 13 | Scottburgh | KwaZulu-Natal | -29,9853; 30,93679 |
| 14 | R66 | KwaZulu-Natal | -29,0166; 31,58311 |
| 15 | N2 | KwaZulu-Natal | -30,0738; 30,86943 |
| 16 | Bendigo | KwaZulu-Natal | -30,6762; 30,50448 |
| 17 | Nelson | KwaZulu-Natal | -31,0518; 30,20742 |
| 18 | Nyalaza | KwaZulu-Natal | -28,2143; 32, 30701 |
| 19 | Park Rynie | KwaZulu-Natal | -30,3039; 30,66676 |
| 20 | Mthunzini | KwaZulu-Natal | -29,0202; 31,59032 |
| 21 | Pietermaritzburg | KwaZulu-Natal | -29.80529; 30,74637 |
| 22 | Salt Rock | KwaZulu-Natal | -29,4786; 31,21786 |
| 23 | Munster | KwaZulu-Natal | -31,0017l 30,24848 |
| 24 | R61 | KwaZulu-Natal | -31,0017; 30,24849 |
| 25 | Umzinto | KwaZulu-Natal | -30,1261; 30,84441 |
| 26 | Durban rail reserve | KwaZulu-Natal | -29,5294; 31,16971 |
| 27 | Wild coast | KwaZulu-Natal | -31,084; 30,18862 |
| 28 | Onrus | Western Cape | -32,3186; 18,40576 |

| | | | |
|---|---|---|---|
| 29 | Magnolias | Western Cape | -33,9449; 18,84668 |
| 30 | Onrus | Western Cape | -34,4071; 19,16236 |
| 31 | Napier | Western Cape | -32,3186; 18,40576 |
| 32 | Biscay road | Eastern Cape | -33,5314; 26,88238 |
| 33 | South down | Eastern Cape | -33,6165; 26,87168 |
| 34 | Word of truth | Eastern Cape | -33,5871; 26,90543 |
| 35 | Wilshire | Eastern Cape | -33,3055; 26,50949 |
| 36 | Cause way | Eastern Cape | -33,5938; 26,88705 |
| 37 | Strand street | Eastern Cape | -33,5897; 26,90413 |
| 38 | Cause way | Eastern Cape | -33,5938; 26,88687 |
| 39 | Word of truth | Eastern Cape | -33,5897; 26,90408 |
| 40 | West road | Eastern Cape | -33,5911; 26,88248 |
| 41 | N2 | Eastern Cape | -29,6261; 31,12441 |
| 42 | Wilshire | Eastern Cape | -33,3168; 26,52654 |
| 43 | Market street | Eastern Cape | -33,3146; 26,53056 |
| 44 | Currie street | Eastern Cape | -33,3021; 26,53455 |
| 45 | York Street | Eastern Cape | -33,3138; 26,5369 |
| 46 | Wilshire | Eastern Cape | -33,3051; 26,50857 |
| 47 | Plumbridge drive | Eastern Cape | -33,2926; 26,52554 |
| 48 | Bhisho road | Eastern Cape | -32,8586; 27,43221 |
| 49 | Highfield | Eastern Cape | -32,9608; 27,9354 |
| 50 | Symtheland road | Eastern Cape | -32,9549; 27,90663 |
| 51 | Kenton road | Eastern Cape | -33,6024; 26,85664 |
| 42HW | HW9 | Big island (Kula), Hawaii | 20,796538; 156,326537 |
| 45FL | IDN4 | Fellsmere, Florida | 27,76718; -80,53387 |

**Table S3.** Primer sequences used to obtain *Schinus terebinthifolia* haplotypes.

| **Primer** | **Sequence(5'-3')** | **Length of obtained sequence (bp)** |
|---|---|---|
| *trnS*[1] | GCCGCTTAGTCCACTCAGC | 844 |
| *trnG*[1] | GAACGAATCACACTTTTACCAC | 844 |

[1]Hamilton 1999

**Table S4.** Primer sequences used in this study and their allelic size range for six microsatellite loci of *Schinus terebinthifolia.*

| Locus | Multiplex | Primer sequence (5'-3') | Fluorescent Dye | No. repeat units | Allele size range (bp) | Source |
|---|---|---|---|---|---|---|
| StAAG13 | A | F:TCACGTTCATGATGCAAAGA<br>R: GATTCCCACGTCAGATTCGT | 6-FAM | CTT(15) | 162-224 | William *et al.* (unpub. data) |
| StAAG14 | A | F:GCAGAATCACACTACTCAGTCACC<br>R: TGGAATGGGTTGGAGGTAGA | VIC | CTT(14) | 157-202 | William *et al.* (unpub. data) |
| StGGT39F | A | F:GACACACCCAAATGACTCACA<br>R: CGGGCAGAATTTGATGAAGA | NED | GGT(9) | 200-258 | William *et al.* (unpub. data) |
| StCTCCTT01 | A | F:TCTCGCATTTCAAGATCACG<br>R: AGCAGTAGGTCGAGGAGGAGT | PET | CTCCTT(6) | 126-176 | William *et al.* (unpub. data) |
| StAAT1 | B | F:AAGGGTGAGAATCTGAAATTTA<br>R: GGCAAACCATTAGTGAGTTTAT | 6-FAM | AAT(21) | 114-192 | William *et al.* (2002, 2005) |
| StAAT16 | B | F:AACAGCCCACCATTTTAACA<br>R: TGGGTAGGTGATGCAGTTCTA | NED | AAT(10) | 152-213 | William *et al.* (2002, 2005) |

**Table S5.** P-values for linkage disequilibrium of each pair of loci across all populations (Fisher's method). Using the Bonferroni correction (*P* < 0.003) there were no significant deviations.

| **Locus pair** | **Chi$^2$** | **df** | ***P*-value** |
|---|---|---|---|
| StAAT1a & StAAT16a | 1.314758 | 8 | 0.995373 |
| StAAT1a & StAAG13 | 12.981777 | 8 | 0.112478 |
| StAAT16a & StAAG13 | 16.168021 | 10 | 0.094923 |
| StAAT1a & StAAG14 | 6.888031 | 8 | 0.548760 |
| StAAT16a & StAAG14 | 23.580228 | 10 | 0.008796 |
| StAAG13 & StAAG14 | 22.299975 | 10 | 0.013647 |
| StAAT1a & StCTCCTT01 | 20.092149 | 8 | 0.009993 |
| StAAT16a & StCTCCTT01 | 4.205602 | 10 | 0.937596 |
| StAAG13 & StCTCCTT01 | 12.773713 | 10 | 0.236600 |
| StAAG14 & StCTCCTT01 | 11.515335 | 10 | 0.318801 |
| StAAT1a & StGGT39 | 10.782476 | 8 | 0.214332 |
| StAAT16a & StGGT39 | 9.498391 | 10 | 0.485545 |
| StAAG13 & StGGT39 | 8.296148 | 10 | 0.599934 |
| StAAG14 & StGGT39 | 4.954195 | 10 | 0.894217 |
| StCTCCTT01 & StGGT39 | 2.015739 | 10 | 0.996218 |

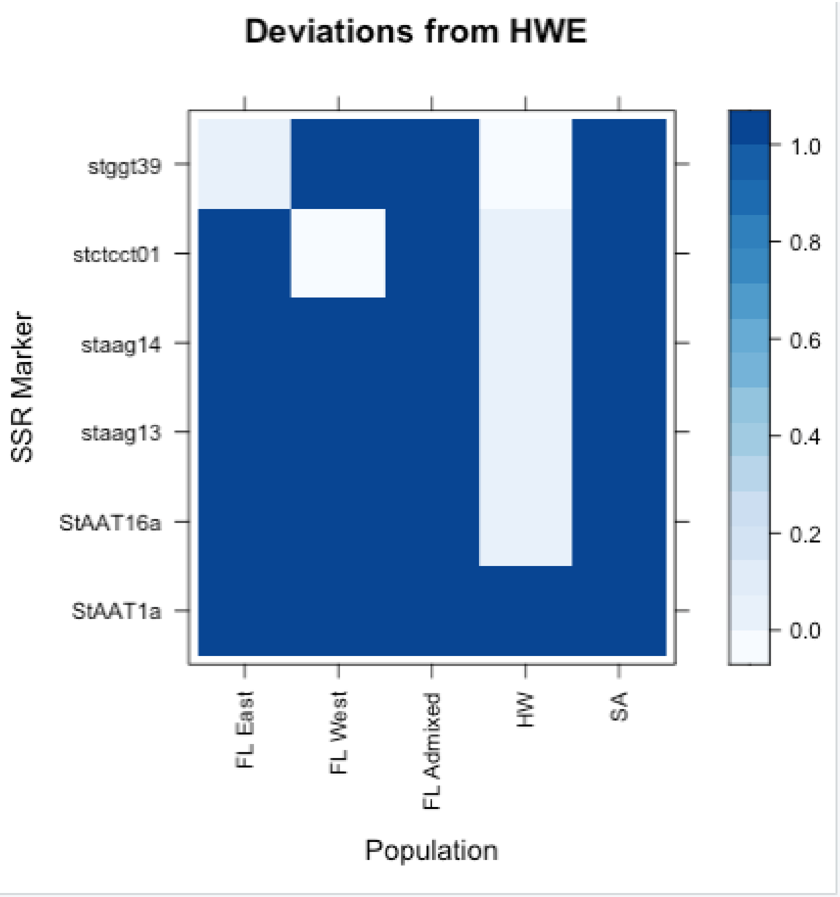


**Figure S1.** Heat map showing *p*-values of deviations from the HWE across populations and markers (*p* <= 0.05). FL East = East Florida, FL West = West Florida, FL Admixed = hybrid populations in Florida, HW = Hawaii, SA = South Africa.

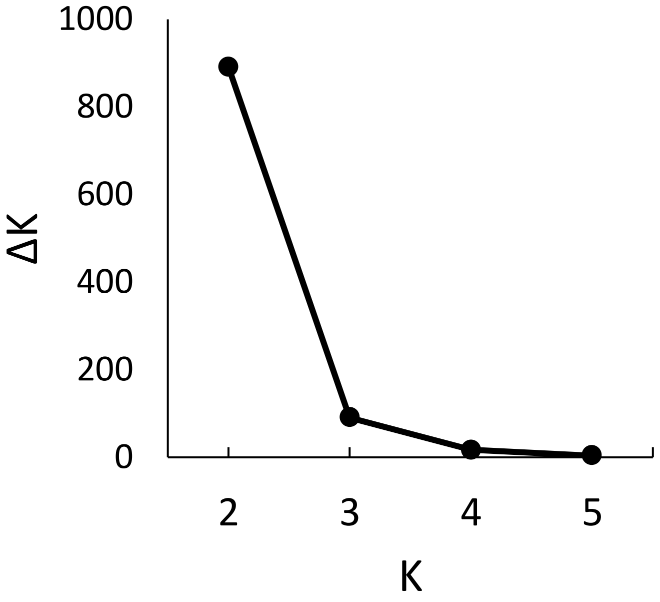


**Figure S2.** Delta K values showing the ideal number of populations as *k* = 2 based on 191 samples of *Schinus terebinthifolia* from South Africa, Hawaii and Florida using six microsatellite primer pairs and the Evanno method implemented in STRUCTURE HARVESTER program according to Earl and von Holdt (2012).

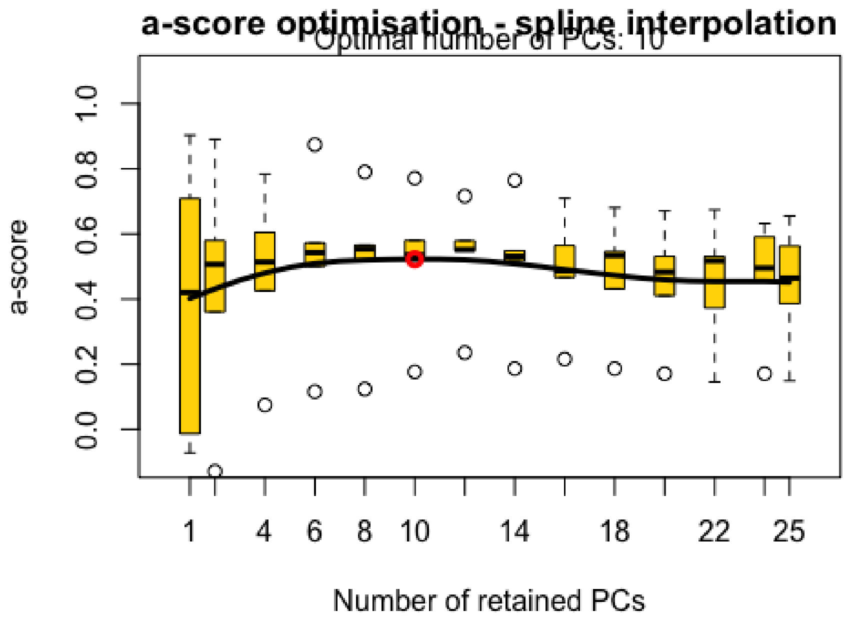


**Figure S3.** The *a-score* optimisation showing the optimum number of retained principal components (PCs) is ten.

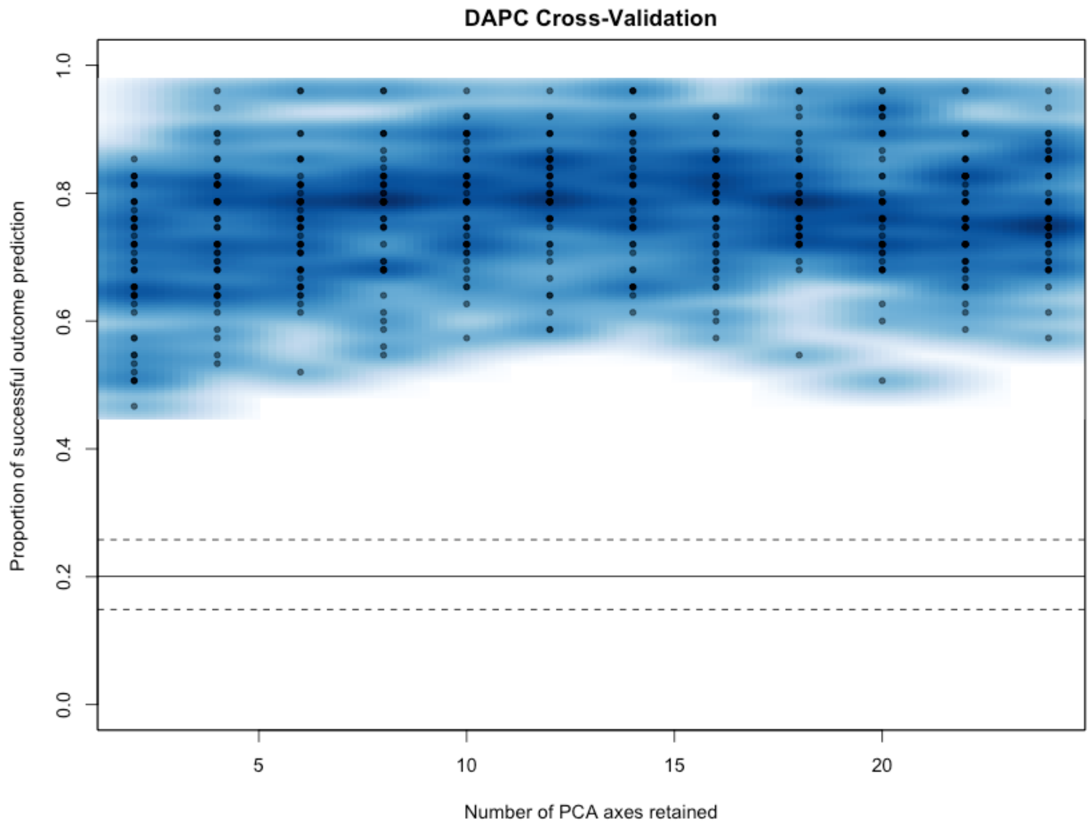


**Figure S4.** The DAPC cross-validation showing the optimum number of retained principal components (PCs) is twenty-five.

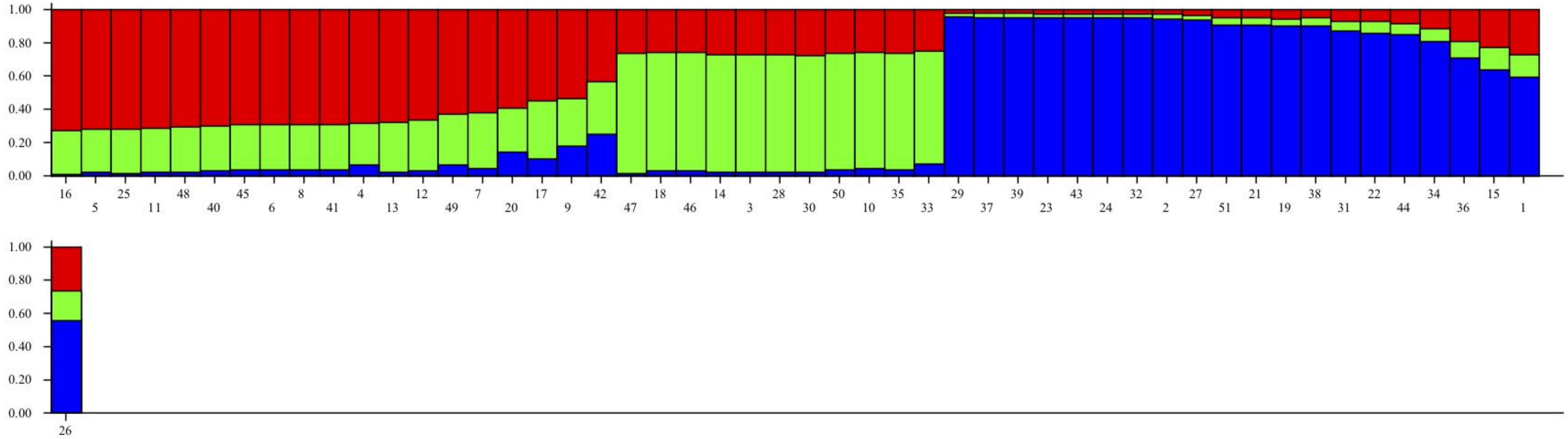


**Figure S5.** Genetic population structure of 51 individuals of *Schinus terebinthifolia* from populations in South Africa, based on Bayesian clustering ana of microsatellite loci with STRUCTURE (Pritchard *et al.* 2000). According to the Evanno method, three populations were inferred (Figure S6). Sample ord according to Q value. The red cluster corresponds to population 1, the green cluster corresponds to population 2 and blue corresponds to population 3. The v in ordinate the shared ancestry according to percentage membership into each population.

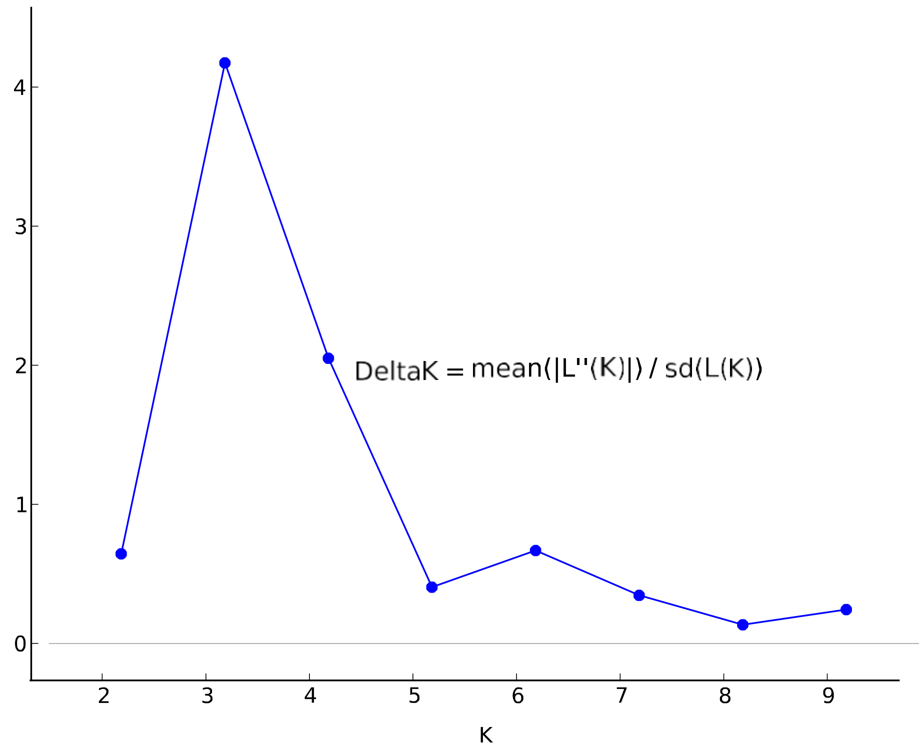


**Figure S6.** Delta K values showing the ideal number of populations as *k* = 3 based on 51 samples of *Schinus terebinthifolia* from South Africa using six microsatellite primer pairs and the Evanno method implemented in STRUCTURE HARVESTER program according to Earl and von Holdt (2012).
